# Impact of coronary bifurcation morphology on wave propagation

**DOI:** 10.1152/ajpheart.00130.2016

**Published:** 2016-07-08

**Authors:** Simone Rivolo, Lucas Hadjilucas, Matthew Sinclair, Pepijn van Horssen, Jeroen van den Wijngaard, Roman Wesolowski, Amedeo Chiribiri, Maria Siebes, Nicolas P. Smith, Jack Lee

**Affiliations:** ^1^Department of Biomedical Engineering, Division of Imaging Sciences and Biomedical Engineering, King's College London, London, United Kingdom, European Union;; ^2^Department of Biomedical Engineering and Physics, Academic Medical Center, University of Amsterdam, Amsterdam, The Netherlands;; ^3^Department of Cardiovascular Imaging, Division of Imaging Sciences and Biomedical Engineering, King's College London, London, United Kingdom, European Union; and; ^4^Faculty of Engineering, The University of Auckland, Auckland, New Zealand

**Keywords:** wave propagation, branching pattern, coronary vasculature, scaling law

## Abstract

The branching pattern of the coronary vasculature is a key determinant of its function and plays a crucial role in shaping the pressure and velocity wave forms measured for clinical diagnosis. However, although multiple scaling laws have been proposed to characterize the branching pattern, the implications they have on wave propagation remain unassessed to date. To bridge this gap, we have developed a new theoretical framework by combining the mathematical formulation of scaling laws with the wave propagation theory in the pulsatile flow regime. This framework was then validated in multiple species using high-resolution cryomicrotome images of porcine, canine, and human coronary networks. Results demonstrate that the forward well-matchedness (no reflection for pressure/flow waves traveling from the coronary stem toward the microcirculation) is a salient feature in the coronary vasculature, and this result remains robust under many scenarios of the underlying pulse wave speed distribution assumed in the network. This result also implies a significant damping of the backward traveling waves, especially for smaller vessels (radius, <0.3 mm). Furthermore, the theoretical prediction of increasing area ratios (ratio between the area of the mother and daughter vessels) in more symmetric bifurcations found in the distal circulation was confirmed by experimental measurements. No differences were observed by clustering the vessel segments in terms of transmurality (from epicardium to endocardium) or perfusion territories (left anterior descending, left circumflex, and right coronary artery).

## Abstract (precis)

*Validation of the proposed theory in multiple species reveals the equivalence between scaling laws and well-matchedness in the vasculature. Moreover, it captures the role of pulsatility in optimal vascular designs. This demonstrates the forward well-matchedness of coronary bifurcations, whereas backward waves are damped asymmetrically at junctions*.

## NEW & NOTEWORTHY

*Validation of the proposed theory in multiple species reveals the equivalence between scaling laws and well-matchedness in the vasculature. Moreover, it captures the role of pulsatility in optimal vascular designs. This demonstrates the forward well-matchedness of coronary bifurcations, whereas backward waves are damped asymmetrically at junctions*.

the branching structure of the coronary network is an important determinant of its function. Understanding the distribution of flow, volume, and resistance and the resulting pressure and velocity wave forms cannot be achieved without accounting for the underlying design of the vascular network. Since the pioneering work of Murray (40, 41), the morphometric relationships between branching vascular segments have been codified into different forms of mathematical relationships, or scaling laws (5, 13, 23, 24, 26, 76, 77, 78). Whereas Murray's original work considered the vascular network as an energy-minimizing structure balancing flow delivery and metabolic costs, subsequent work has explored uniform wall shear stress (24, 76), myocardial mass (5), vascular volume, and cumulative lengths (23, 76) as determinants underlying the design principles.

The current leading theory in vascular network design principles is arguably the volume-scaling law (HK law) (23, 24), which based on a set of biophysical assumptions, coupled cumulative vessel volume and length with the vessel diameter. This approach produced equivalent results to an earlier minimum-energy model (76) that generalized Murray's formulation from a single bifurcation to the entire arterial network. The discrepancies that were found among different vascular beds were explained on the basis of their varying metabolic-to-viscous power dissipation ratios (26).

Quantitatively, these scaling laws serve a useful role in interpreting angiographic data in the context of growth (4), disease, and interspecies comparison (26) in uncovering implied relationships between parameters (56) and estimating the distal flow resistance in myocardial blood flow (e.g., for computational fluid dynamics assessment of fractional flow reserve) (64).

On the other hand, the branching parameters measured in real vascular networks exhibit a large scatter (46, 77). Moreover, fitted power law exponents reported in the literature vary significantly depending on the range of vessel diameters being analyzed (67). This heterogeneity had been characterized as possessing features of multifractality using human coronary data, leading to a speculation that such a property may endow the tree with an enhanced ability to distribute impedances along its structure (78). The existing scaling laws provide no explanation for the origin of such heterogeneity. Furthermore, there is evidence that the theoretical framework of scaling laws leads to a shallow optimum—that is to say, the cost of departure from the power law relationship is minimal over a very broad range of exponents (57). Combined with high heterogeneity, it undermines the significance of the putative design principles proposed.

Aside from the physical organization of the vessel segments, pulsatility in flow is also a central feature that is strongly characteristic in the coronary circulation in both large (36, 55) and small vessels (66), in that the magnitude of the pulsatile component is not necessarily dominated by the steady component. Surprisingly, to date, investigations of coronary network scaling laws have been conducted largely independent of the wave phenomena. This is surprising given that it is the generation, propagation, and reflection of pulse waves that shape the pressure and velocity dynamics throughout the network. In particular, the rhythmic ejection of blood by the left ventricle and the cyclic myocardial contractions that squeeze the embedded vasculature are responsible for the proximal and distal generation of pressure and flow waves. These generated waves then propagate forward (from the coronary root toward the microcirculation) and backward throughout the network, where at each bifurcation encountered, partial wave reflections may alter the wave form shapes. The amplitude of the pulse wave measured at any point in the network therefore carries an imprint of the interaction the propagating wave has undergone with the underlying network structure.

With newer research tools such as wave intensity analysis (WIA) gaining momentum in the clinical arena (8, 62, 63), the clinical diagnostic potential of altered arterial pulse waves in disease has received much attention in recent investigations (7–9, 55). Specifically, cumulative energy (area of the waves) and peak energy (peak of the waves) have been used in clinical settings to enhance our understanding of various disease processes affecting the coronary vasculature [e.g., aortic stenosis (7), left ventricular hypertrophy (8)] and to define the effect of various interventions [e.g., biventricular pacing (34), intra-aortic balloon therapy (10)]. Most recently, the demonstrated prognostic benefit of WIA in the setting of acute myocardial infarction has enhanced prediction of long-term myocardial recovery (9). However, although it is known that left ventricular ejection/suction is the principal factor that generates forward-traveling waves, the main mechanisms underlying backward-traveling waves have yet to be elucidated, and confounds the conclusion derived by applying coronary WIA. Specifically, it is proposed that myocardial compression/expansion on intramural vessels and diastolic reduction of resistance at the microcirculatory level are the two main contributors underlying the backward wave origin (8, 55). However, which of the two is the dominant mechanism and how this dominance varies depending on transmural location and vessel size are still under debate.

At present little is known about how the branching structure of the coronary vasculature is linked with the observed wave propagation behavior. A rare study, and perhaps the earliest work on this subject (1), was the first to propose that the branching structure and mechanical properties of the coronary vessels larger than 0.5 mm in diameter are well matched so as to facilitate the forward-traveling waves to traverse a junction without being significantly dampened. The presented experimental evidence compared well with their theoretically optimal branching structure. However, that theory was based on an assumption of uniform distensibility in branching vessels, which has not been confirmed for consecutive generations of a given vascular network. That work, therefore, as with many other investigations of arterial wave propagation, suffered from difficulties in determining pulse wave speed (PWS) and thus relied on extrapolation of results measured in larger segments. Accurate measurement of coronary PWS is a challenge that persists even today, and there is a large scatter in the values employed in the literature ranging from 5–10 m/s in earlier studies (1, 77) to higher values such as 15–25 m/s, estimated by more recent ComboWire measurements (48, 50). In the analysis below, we show that evoking the theory of scaling law can help to overcome this experimental difficulty.

In short, the existing scaling laws capture well the broad behavior of the vascular branching pattern in an averaged sense. Heterogeneity, however, is better addressed by assessing the wave reflections (and the encompassing theory) because they are inherently a local phenomenon. As demonstrated herein, the application of scaling laws to interventionally relevant epicardial segments results in a large spread that cannot be explained by the existing theory. By unifying the theoretical framework of the scaling law with those of pulsatility and wave propagation, we show that phasic aspects of coronary flow—which after all are one of its defining characteristics—are an indispensable determinant of coronary structural design that can contribute to the observed heterogeneity. Accordingly, what we propose here is not a new scaling law, but a generalized framework by which to elucidate vascular branching structures.

The rest of the paper is organized as follows: we begin by recapitulating the theories of scaling laws and wave reflection in vascular networks before developing the new unified model of the organization of branching structure under pulsatile flow. Following that, the physiological range of Womersley numbers is considered to incorporate the frequency-domain behavior into the analysis. Experimental validation comprising high-resolution imaging and segmentation of coronary networks from several species are then described and applied to the proposed theory. Finally, model results are applied to the new and existing experimental data to explain the spread, to revisit the well-matchedness hypothesis, and to investigate regional and scale-specific differences in coronary branching patterns. In addition, existing scaling laws that were constructed in the absence of pulsatile flow are reassessed to show that each proposed law implies a specific branching pattern, which has not been evaluated to date in terms of resulting wave propagation.

## METHODS

We begin with a brief presentation of the mathematical background of the scaling laws regarding the coronary branching pattern (23, 24, 40, 41, 78). Subsequently, the one-dimensional (1D) blood flow theory is introduced along with the concepts underlying wave reflection at bifurcations. These two theoretical frameworks are then combined by incorporating the effect of flow pulsatility (using Womersley's approach) into the analysis. Finally the experimental protocol and imaging processing pipeline employed for coronary anatomical reconstruction are described, followed by the model validation.

### Theory of Scaling Law at Bifurcations

A scaling law is an analytic formulation describing morphometric relationships, often parameterized by the vessel lengths, diameters, and arterial volume between a feeding segment and the perfused subtree (13, 24, 78). Because the effect of a scaling law on wave reflection at bifurcations is of primary interest in this study, we focus on a specific aspect of the scaling laws: the relationship between the mother vessel diameter (*d*_*m*_) and the diameter of the daughter vessels (*d*_*d*1_, *d*_*d*2_) at each bifurcation. This relationship can be written in a generic form in terms of either diameter or cross-sectional area as

(1) $${\left(\frac{d_{d_{1}}}{d_{m}}\right)}^{\tau }+{\left(\frac{d_{d_{2}}}{d_{m}}\right)}^{\tau }=1$$

(2) $${\left(\frac{A_{d_{1}}}{A_{m}}\right)}^{\frac{\tau }{2}}+{\left(\frac{A_{d_{2}}}{A_{m}}\right)}^{\frac{\tau }{2}}=1$$

where the subscripts *m*, *d1*, and *d2* denote the mother and daughter vessels, respectively. τ is the scaling parameter that characterizes the different scaling laws (13). The well-known Murray's law was the first scaling law proposed, where τ = 3 was derived by minimizing the combined viscous power dissipation and the metabolic power expenditure across the vascular network (40, 41). Subsequent investigations (27, 76) demonstrated that the main limitation of Murray's law was that it considered each bifurcation in isolation instead of being part of a complete network and proposed successive improvements (based on the minimization of the cost of fluid conduction and fluid metabolism) culminating in the well-established HK law, with an exponent τ = 7/3 (23, 24). In the analysis below, these two laws are considered in detail.

### One-Dimensional Blood Flow Theory

The mathematical background of the 1D blood flow formulation has been extensively described in the literature (14, 36, 37, 58, 59). Importantly, the forward and backward wave reflection coefficients at a bifurcation derive from the following system of conservation equations (mass and momentum) in three variables: cross-sectional area, pressure, and velocity (*A*, p, *v*):

(3) $$\frac{\partial A}{\partial t}+\frac{\partial \left(Av\right)}{\partial x}=0$$

(4) $$\frac{\partial v}{\partial t}+\alpha v\frac{\partial v}{\partial x}+\frac{1}{\rho }\frac{\partial p}{\partial x}=-\kappa v.$$

where α is a nondimensional correction factor for momentum flux, ρ is the blood density, and κ represents the viscous resistance of the flow per unit length of vessel (60). This system is closed by a constitutive law relating pressure to area, derived from an elastic linear shell model (2):

(5) $$p\left(x,t\right)=\beta \left(x\right)\left(\sqrt{A\left(x,t\right)}-\sqrt{A_{0}\left(x\right)}\right)$$

(6) $$\beta \left(x\right)=\frac{\sqrt{\pi }E\left(x\right)h\left(x\right)}{\left(1-v^{2}\right)A_{0}\left(x\right)}$$

Here, *A*_0_ indicates the reference area and β indicates the vessel material properties, which in turn are dependent on the Young's modulus *E*, the vessel thickness *h*, and Poisson's ratio *ν*, which is usually taken as 0.5 because biological tissue is nearly incompressible (14, 59).

The PWS, denoted as *c*, which is the speed at which the wave front propagates through the vessel, is derived from the characteristic analysis of the system of *Eqs. 3* and *4*, as shown in Sherwin et al. (59):

(7) $$c=\sqrt{\frac{\beta }{2p}A^{\frac{1}{4}}}.$$

The formulation outlined above for a single blood vessel can be then extended to a vascular network by imposing suitable coupling conditions at the vessel junctions (58, 59). A common approach is to represent junctions as a single point and to disregard the effect of the branching angles and momentum loss, because it has been shown that they play only a minor role in wave propagation in the physiological range of pressure and velocity in coronary vessels (14).

### Wave Transmission Theory

In this work we employ a linearized wave transmission regime. Although this approximation is most accurate when the variation in the underlying hemodynamics is small, the tractability of the linear analysis can offer several important insights as demonstrated in Sherwin et al. (59). In this theory, the reflection coefficient *R*_*f*_ at a bifurcation is defined as the ratio of the amplitude of the reflected and incident waves:

(8) $$R_{f}=\frac{\frac{1}{z_{0,m}}-\frac{1}{z_{0,d_{1}}}-\frac{1}{z_{0,d_{2}}}}{\frac{1}{z_{0,m}}+\frac{1}{z_{0,d_{1}}}+\frac{1}{z_{0,d_{2}}}}$$

where the characteristic impedance of a vessel, *Z*_0_, relates the velocity or flow of a wave with the applied pressure and is defined as:

(9) $$Z_{0}=\frac{\rho c_{0}}{A_{0}}$$

Consequently, the reflection coefficient, *R*_*f*_, can be rewritten in terms of cross-sectional area and PWS as:

(10) $$R_{f}=\frac{\frac{A_{0,m}}{c_{0,m}}-\frac{A_{0,d_{1}}}{c_{0,d_{1}}}-\frac{A_{0,d_{2}}}{c_{0,d_{2}}}}{\frac{A_{0,m}}{c_{0,m}}+\frac{A_{0,d_{1}}}{c_{0,d_{1}}}+\frac{A_{0,d_{2}}}{c_{0,d_{2}}}}$$

under the reasonable assumption of constant blood density. It is important to highlight that from *Eq. 7*, the PWS in the reference configuration is:

(11) $$c_{0}=\sqrt{\frac{\beta *}{2\rho }}A_{0}^{-\frac{1}{4}}$$

(12) $$\beta^{*}=\beta A_{0}$$

Note that commonly in the definition of β (*Eq. 6*) the term *A*_0_ is included. However, because in this analysis *A* = *A*_0_ as a result of linearization, it is preferable to introduce an area-independent measure, β*. The backward reflection coefficients for the daughter vessels, *R*_*d*1_, *R*_*d*2_, can be analogously derived by swapping in *Eq. 10* the term related to the mother vessel $\frac{A_{0,m}}{c_{0,m}}$ with $\frac{A_{0,d_{1}}}{c_{0,d_{1}}}$ or $\frac{A_{0,d_{2}}}{c_{0,d_{2}}}$.

### Unified Framework

**Fig. 1.**
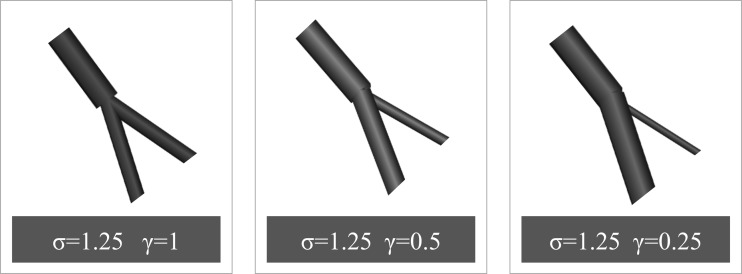
Three examples of bifurcations with the same area ratio, σ, but different symmetry ratios, γ.

To explore their intrinsic relationship, the scaling law formulation and the wave transmission theory have to be brought into a common framework. This can be achieved by recasting the mathematical formulation of the two frameworks in terms of the area ratio, σ, and the symmetry ratio, γ (see Fig. 1), defined as

(13) $$\sigma =\frac{A_{d_{1}}+A_{d_{2}}}{A_{m}}$$

(14) $$\gamma =\frac{A_{d_{2}}}{A_{d_{1}}}\;\;\;A_{d_{2}}<A_{d_{1}},0\leq \gamma \leq 1$$

Here onward, σ and γ are used as the main parameters in our model derivation. Note that γ = 0 implies no bifurcation, whereas γ = 1 describes a perfectly symmetrical bifurcation.

Starting from the generic form of a scaling law (see *Theory of Scaling Law at Bifurcations*) and considering that $\frac{\sigma }{1+\gamma }=\frac{A_{d_{1}}}{A_{m}}$, *Eq. 2* can be rewritten in terms of σ, γ as:

(15) $${\left(\frac{\sigma }{1+\gamma }\right)}^{\frac{\tau }{2}}\left(1+\gamma^{\frac{\tau }{2}}\right)=1.$$

A closed form expression of σ in terms of γ can be then easily derived as:

(16) $$\sigma =\frac{1+\gamma }{{\left(1+\gamma^{\frac{\tau }{2}}\right)}^{\frac{2.}{\tau }}}$$

In the wave transmission theory, assuming without loss of generality that *A* ≈ *c*^ξ^, the forward reflection coefficient can be rewritten as:

(17) $$R_{f}=\frac{1-{\left(\frac{\sigma }{1+\gamma }\right)}^{\frac{\tau }{2}}\left(1+\gamma^{\frac{\tau }{2}}\right)}{1+{\left(\frac{\sigma }{1+\gamma }\right)}^{\frac{\tau }{2}}\left(1+\gamma^{\frac{\tau }{2}}\right)}$$

after the variable substitution $1-\xi =\frac{\tau }{2}$. It is then evident that imposing *R*_*f*_ = 0 in *Eq. 17*, thus implying no reflections for forward-traveling waves, provides the theoretical σ-γ relationship of *Eq. 16*, which has been derived from the scaling law framework. This is a crucial point of the analysis and will be extensively discussed in results. The backward reflection coefficients *R*_*d*1_, *R*_*d*2_ can be rewritten in terms of σ and γ following the same steps as presented above.

### Effect of Pulsatility

The derivation above has unified the framework of scaling laws with the wave transmission theory and enables the link between branching structure (as characterized by a specific scaling law) and wave propagation to be investigated (*Eqs. 16* and *17*). However, this result has been derived under the assumption of steady conditions, thus neglecting the effect of flow pulsatility. In this section we apply Womersley's analysis to generalize our derivation. To date, the effect that pulsatility has on the forward and backward reflection coefficients at each bifurcation has been evaluated only in systemic arteries where the flow regime is different compared with the coronary vasculature (3).

Specifically, the formulation presented above (*Eqs. 9* and *10*) regarding the reflection coefficients at a bifurcation is commonly used in the literature. However, it is important to recall that it is valid only for Womersley's number, α > 10 (3, 46, 70, 71), where, as explained by Papageorgiou and Jones (46), the characteristic impedance, *Z*_0_, can be adequately simplified into $Z_{0}=\frac{\rho c_{0}}{A_{0}}$. Because lower Womersley numbers are typical in the coronary vasculature (28), the more general form of the characteristic impedance *Z*_0_ [described in Brown (3), Nichols et al. (44), and Papageorgiou and Jones (46)] should be considered when addressing the coronary circulation:

(18) $$Z_{0}=\frac{\rho c_{0}}{A_{0}}\frac{1}{\sqrt{M_{0}^{1}\left(1-v^{2}\right)}}e^{-\frac{i\epsilon_{0}}{2}}$$

where $M_{0}^{\prime }$ and ϵ_0_ are functions of the Womersley number, α (70, 71). These functions were introduced by Womersley (70) to obtain a more compact expression of the velocity profile in pulsatile flow (44). A full description and significance of each of these variables are briefly outlined in the appendix, and more extensively in the references (44, 70, 71). Essentially, in this study the values of $M_{0}^{\prime }$ and ϵ_0_ for different values of α are derived from those tabulated by Womersley (70).

Therefore, following the analysis presented in Brown (3), for a given Womersley number in the mother vessel, α_*m*_, the corresponding values in the daughter vessels can be calculated as

(19) $$\alpha_{d_{1}}=\alpha_{m}\sqrt{\frac{\sigma }{1+\gamma }}$$

(20) $$\alpha_{d_{2}}=\alpha_{m}\sqrt{\frac{\sigma \gamma }{1+\gamma }}$$

This reveals that at each junction, α may vary heterogeneously from segment to segment as a function of the area ratio and symmetry ratio. The reflection coefficient, *R*_*f*_, in *Eq. 17* can be rewritten, following the same steps, as

(21) $$R_{f}=\frac{1-D}{1+D}$$

where

(22) $$D={\left(\frac{\sigma }{1+\gamma }\right)}^{\frac{\tau }{2}}\left(\sqrt{\frac{M_{d_{1}}}{M_{m}}}e^{-\frac{i\left(\epsilon_{d_{1}}-\epsilon_{m}\right)}{2}}+\gamma^{\frac{\tau }{2}}\sqrt{\frac{M_{d_{2}}}{M_{m}}}e^{-\frac{i\left(\epsilon_{d_{2}}-\epsilon_{m}\right)}{2}}\right)$$

The effect of varying Womersley number on wave propagation can now be assessed. It is important to highlight that *D* is a complex number, but no reflections occur when *Re*(*D*) = 1 (3). The σ-γ relationship then becomes

(23) $$\sigma =\frac{1+\gamma }{ℜ({\left(\sqrt{\frac{M_{d_{1}}}{M_{m}}}e^{\frac{-i\left(\epsilon_{d_{1}}-\epsilon_{m}\right)}{2}}+\gamma^{\frac{\tau }{2}}\sqrt{\frac{M_{d_{2}}}{M_{m}}}e^{\frac{-i\left(\epsilon_{d_{2}}-\epsilon_{m}\right)}{2}}\right)}^{\frac{2}{\tau }}).}$$

### Coronary Anatomy Reconstruction

#### Experimental procedure.

Optical fluorescence cryomicrotome imaging is a modality that allows high-resolution, large-volume acquisition of vascular tissue injected with fluorescent contrast agents (35, 61). The vascular casting experimental procedure has been presented in previous publications (16, 25, 48, 67, 69b), where further details can be found. The three porcine cryomicrotome vasculature data sets in this work were obtained from experiments conducted with approval by the U.K. Home Office in accordance with the UK Animals (Scientific Procedures) Act of 1986 (under license no. 7002723) and in compliance with the World Medical Association Declaration of Helsinki regarding ethical conduct of reserch involving animals. The canine heart was obtained from experiments carried out according to a protocol approved by the Institutional Animal Care and Use Committee of the University of Utrecht in The Netherlands. The human heart was obtained postmortem from the Department of Pathology at the Academic Medical Center (University of Amsterdam) with written consent from the patient's relatives for organ donation, and the code of good practice for use of human tissue in The Netherlands was observed. The subject was a man in his 40s, with the cause of death unrelated to cardiovascular disease. The hearts were surgically extracted and suspended, and flushed with adenosine-loaded (100 μg/l) PBS. The excised hearts were in a diastolic state at the time of coronary filling. The porcine hearts were then perfused with Batson's No. 17 vascular casting resin (Polysciences, Germany) containing fluorescent Potomac yellow dye at an average arterial pressure (90 mmHg). Ultraviolet blue dye (VasQtec, Zurich, Switzerland) was added to the casting resin for the human and canine hearts. The injected casts were then allowed to harden for up to 24 h at ambient temperature. Following this, the hearts were immersed and the ventricular chambers were filled with carboxymethylcellulose sodium solvent (Brunschwig Chemie, The Netherlands) and Indian ink (Royal Talens, The Netherlands) mixture, and stored at −20°C. The samples were then transferred to the cryomicrotome and sequentially sliced and imaged using fluorescent optical surface imaging (16, 69b). The image stack of each vasculature was then imported into our custom-developed automatic vascular extraction software, and a geometrical representation graph was obtained. The three porcine hearts processed were resampled to isotropic voxel sizes of 58, 54, and 53 μm, respectively, whereas the original voxel sizes were half these dimensions. The voxel sizes of the human heart and the canine heart were 58 and 50 μm, respectively.

#### Automatic vascular extraction.

**Fig. 2.**
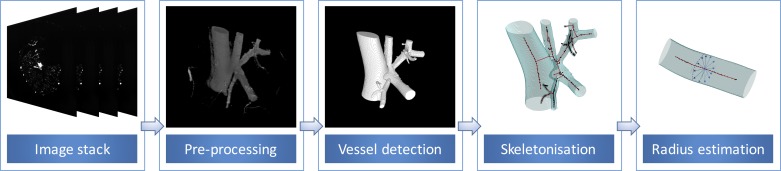
The main steps constituting our vascular extraction software.

The main steps behind the custom 3D vessel segmentation pipeline are summarized in Fig. 2. Note that the current work utilizes an enhanced implementation, compared with the one presented in Goyal et al. (16).

To improve the quality of the original image stack (4,000 × 4,000 × 2,000 image slices), image restoration techniques were initially applied, including deconvolution algorithms to tackle blurring (16). Subsequently, the range of vessel sizes typical of the coronary vasculature were detected separately by applying multiscale Hessian-based filters and then combined (15). In addition, the multiscale vesselness descriptor was expanded by complementing it with addition of the Sato vesselness measure (53). Centerline extraction was then performed by applying the medial surface/axis thinning algorithm on the binarized volume (39). Subsequently, the vessel diameters were estimated and subvoxel adjustment of the medial points were achieved by applying the Rayburst sampling algorithm (49) combined with a 3D core that follows the vessel centerline, providing higher accuracy than the sphere-fitting algorithm (16). The quality of the extracted vasculature network was further enhanced by a series of algorithms that prune out nonphysiological structures across the vasculature, typically caused by cast leakage.

### Validation of the Unified Framework

**Fig. 3.**
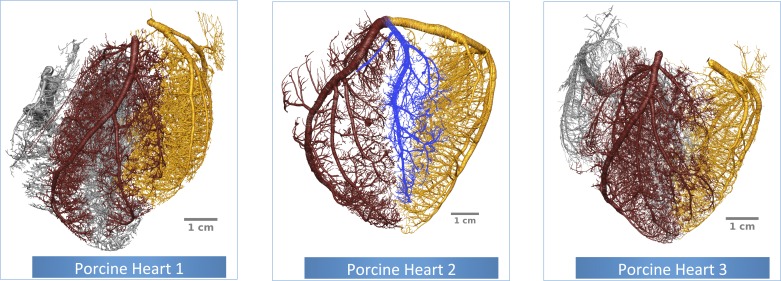
The reconstructed ex vivo porcine coronary vasculatures used for this study. The left anterior descending artery (LAD), left circumflex artery (LCx), right coronary artery (RCA), and marginal artery subtrees are highlighted in brown, gold, silver, and blue, respectively.

The newly developed theoretical framework presented above (see *Unified Framework* and *Effect of Pulsatility*) is validated using anatomical measurements obtained from three high-resolution cryomicrotome volume images of ex vivo porcine coronary vasculature (Fig. 3). These vascular trees provide anatomical measurements of ≈80,000 bifurcations with a radius range of 1.6–50 μm. The data were obtained by a custom-developed experimental setup and vascular extraction pipeline that were described in the previous section.

**Fig. 4.**
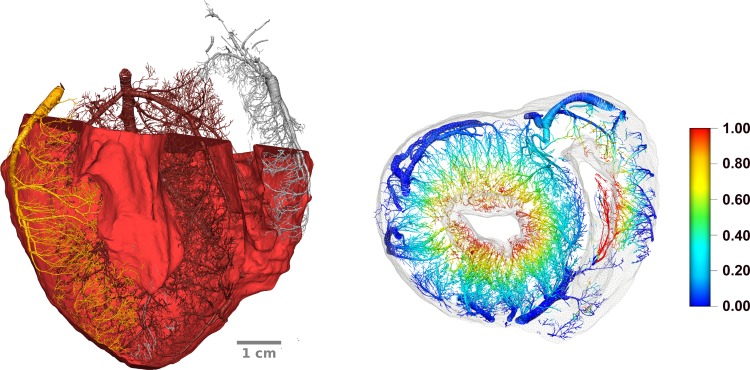
*A*: a long-axis section of the porcine vasculature with the surrounding myocardial wall. *B*: a short-axis slice of the vasculature with the ventricular mesh in light gray. The color spectrum represents the epi-endo mapping calculated using the method detailed in the text.

The high-resolution cryomicrotome volume images provide sufficient anatomical detail to be analyzed both from a hierarchical point of view (radius of the mother vessel) as well as separating the territories perfused by the large epicardial vessels [left anterior descending artery (LAD), left circumflex artery (LCx), and right coronary artery (RCA)]. In addition, for each of the porcine vascular networks (see Fig. 4), the myocardium (comprising both the left and right ventricles) has been manually segmented [using ImageJ software (54)], and tetrahedralized [using CGAL (65)]. Following this step, the Laplace equation has been solved with Dirichlet boundary conditions comprising 0 and 1 as boundary conditions on the epicardial and endocardial surfaces to obtain a linear transmural gradient through the myocardial wall. The transmural depth was then assigned to each bifurcation using a kd-tree based search algorithm (to find the k nodes of the ventricular mesh closest to a vascular node) with *k* = 10, thus offering an additional classification of the extracted coronary networks in terms of transmurality.

From these data, the statistical distributions of both σ and γ were initially calculated. The spatial distributions of the area and symmetry ratios ware then investigated to assess whether these quantities correlate with different vessel scales, perfused territories, or transmural location. The σ-γ theoretical relationship (*Eqs. 16–23*) for the different scaling laws considered (Murray's law and the HK law) was then compared with the measurements, including the effect of varying Womersley number. Following this analysis, the forward and backward reflection coefficients (*R*_*f*_, *R*_*d*1_, and *R*_*d*2_) were calculated directly from the experimental data using *Eq. 10*, and compared with the theoretical predictions. However, note that *Eq. 10* depends on knowledge of both anatomical measurements and the PWS (or alternatively the material properties, β*) in each vessel. Thus the distribution of the PWS (or β*) across the vasculature had to be hypothesized because it cannot be experimentally measured in every vessel at present. Therefore, in the following, we examine several common hypotheses employed in the literature. Two simpler assumptions are to impose a constant uniform PWS (37) (which implies decreasing β* distally) or uniform material property (implying an increase in the PWS distally) (42) throughout the network. A third common approach (43, 69) relies on an empirical relationship described by Olufsen (45), based on measurements from mostly the systemic circulation:

(24) $$\frac{Eh}{r_{0}}=k_{1}e^{k_{2}r_{0}}+k_{3}$$

where *k*_1_ = 2 × 10^7^ g·s^−2^·cm^−1^, *k*_2_ = −22.53 cm^−1^, and *k*_3_ = 8.65 × 10^5^ g·s^−2^·cm^−1^. Each of these three approaches was applied to the extracted porcine coronary vasculatures to calculate *R*_*f*_, *R*_*d*1_, and *R*_*d*2_ at each bifurcation.

Finally, the results derived from porcine vasculatures were compared with those from the other species (one human and one canine vasculature) to investigate possible interspecies variation.

## RESULTS

### Unified Framework

The theoretical analysis described in the previous section (*Eqs. 14–17*) has multiple implications. To our knowledge, for the first time it demonstrates the equivalence between the adherence of a vascular network to a particular scaling law and the well-matchedness of its bifurcations, thereby closing the loop between vascular design and wave propagation dynamics. Moreover, if well-matchedness is assumed, then an σ-γ closed-form analytical relationship is derived (*Eq. 16*) and can be used for validation, because it depends solely on the anatomical measurements without requiring knowledge of the PWS or the material properties in each segment, which are not currently experimentally measurable.

**Fig. 5.**
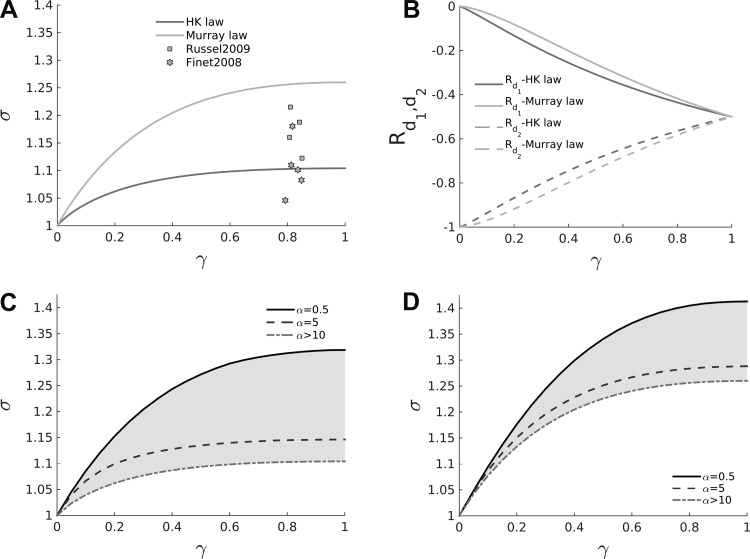
*A*: the σ-γ relationship for the two scaling laws considered. Murray's law predicts higher area ratios for a given symmetry ratio than the HK law. *B*: reflection coefficients for backward-traveling waves for both the larger and smaller daughter vessels. It is evident how asymmetrical bifurcations (γ < 0.5) strongly disadvantage backward-wave propagation (highly negative reflection coefficient) in the smaller daughter vessel. *C* and *D*: shaded regions span the range of varying Womersley number on the σ-γ relationship (*Eq. 23*) for both the HK law (*C*) and Murray's law (*D*) [α > 10 represents the case when the effect of pulsatility is disregarded (*Eq. 16*)]. For lower α the theory predicts that the well-matchedness hypothesis is fulfilled at higher area ratios for a given symmetry ratio.

In Fig. 5*A*, the theoretical σ-γ relationships are displayed for the two different scaling laws being considered, along with experimental data points measured from human epicardial coronary vessels. If well-matchedness is assumed a priori, then both Murray's law and the HK law predict that more symmetrical bifurcations (γ > 0.5) would exhibit higher area ratios, which can be tested against experimental measurements. It can be seen that Murray's law predicts higher area ratios for a given symmetry ratio compared with the HK law, and that neither law adequately captures the spread of the data points. Furthermore, the measurements provided by Finet et al. (12) and Russell et al. (52) cover a relatively small area ratio range (γ ≈ 0.8) and are limited to large vessels (*d* > 2 mm), which motivates the need for further experimental investigation.

The impact of Womersley number, α, on the σ-γ relationship is displayed for the HK law (Fig. 5*C*) and Murray's law (Fig. 5*D*). The shaded regions represent the area spanned by the relationship for each law, when α is varied within a physiological range. The Womersley numbers that are typical of the coronary vasculature being considered are 0.01 ≤ α ≤ 10 following Kassab et al. (28). Similarly to systemic arteries (3), the theory predicts that for a given symmetry ratio, a decrease in the Womersley number causes an increase in the area ratio necessary to achieve well-matchedness (i.e., for a given symmetry ratio, smaller vessels should exhibit higher area ratios). Furthermore, this has an important implication for the validation of the theoretical framework. Because the precise Womersley number, α, for each bifurcation is unknown but may vary heterogeneously in accordance with *Eqs. 19* and *20* for each scaling law, the σ-γ measurements derived from the porcine vasculatures should not be compared with the σ-γ relationship obtained for a specific α. More correctly, the area ratio and symmetry ratio measurements should be compared with the area spanned by the σ-γ relationship of a specific scaling law for the physiological range 0.01 ≤ α ≤ 10. This is the approach pursued below.

Finally, in Fig. 5*B*, the reflection coefficients for backward-traveling waves (*R*_*d*1_, *R*_*d*2_) in the two daughter vessels are displayed for both the HK law and Murray's law. It is important to highlight that the relationships displayed are valid under the assumption of forward well-matchedness. In symmetrical bifurcations, backward wave transmission is impeded (*R*_*d*1_ ≈ *R*_*d*2_) in both daughter vessels to a similar degree, whereas in more asymmetrical bifurcations, backward wave transmission through the junction is favored (*R*_*d*1_ < *R*_*d*2_) from the larger daughter vessel at the expense of the smaller daughter vessel. Therefore, from the point of backward waves, a higher degree of asymmetry at bifurcations is more advantageous because it allows backward-traveling waves to carry their information farther. Finally, the theory correctly predicts no reflection in the larger daughter vessel and full reflection in the smaller one when γ ≈ 0 (no bifurcation).

### Vascular Extraction Results

**Fig. 6.**
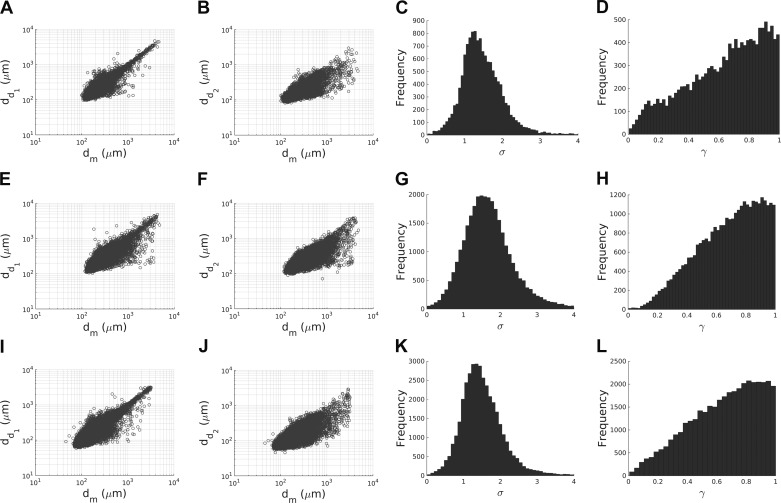
For each porcine vasculatures analyzed (*A*–*D*: specimen 1; *E*–*H*: specimen 2; *I*–*L*: specimen 3), the relationships between the mother diameter (*d*_*m*_) and the large (*d*_*d*1_) and small (*d*_*d*2_) daughter vessel diameters are shown using a log-log scale in the first (*A*, *E*, and *I*) and second (*B*, *F*, and *J*) columns, respectively. The trend is in good agreement with the literature (65). The histograms of the area ratio, σ, and symmetry ratio, γ, are presented in the third (*C*, *G*, and *K*) and fourth (*D*, *H*, and *L*) columns. The distribution is qualitatively consistent between vasculatures.

Before describing data analysis, here we compare the reliability of the vasculature extraction pipeline against that found in previous literature. The percentage of bifurcations and *n* furcations (with *n* > 2) in each extracted porcine vasculatures is ≈95% and ≈4%, respectively, which is in good agreement with data reported by Kassab et al. (29). Trifurcations are excluded in the subsequent analysis because of their limited number in the coronary vasculature (29, 67). Moreover, as can be seen in Fig. 6, the relationship between the diameter of the mother and daughter vessels in each network compare well with that reported by VanBavel and Spaan (67), demonstrating the reliability of the radius estimation step in the vascular extraction pipeline. Finally, since the statistical distributions of the area ratio and symmetry ratio in Fig. 6 (right two columns) agree well with data reported by VanBavel and Spaan (67) and are qualitatively consistent between samples, the data sets were merged for the remainder of the analysis.

### Distribution of σ-γ Across the Vasculature

As a precursor to full validation, variations in area and symmetry ratios across the coronary vasculature were assessed because they are the key variables governing wave propagation in the network. To do so, the data from the three porcine vascular networks were combined and divided into clusters according to either the mother radius, the perfused territory (LAD/LCx/RCA), or the transmural location (3 equal layers).

**Fig. 7.**
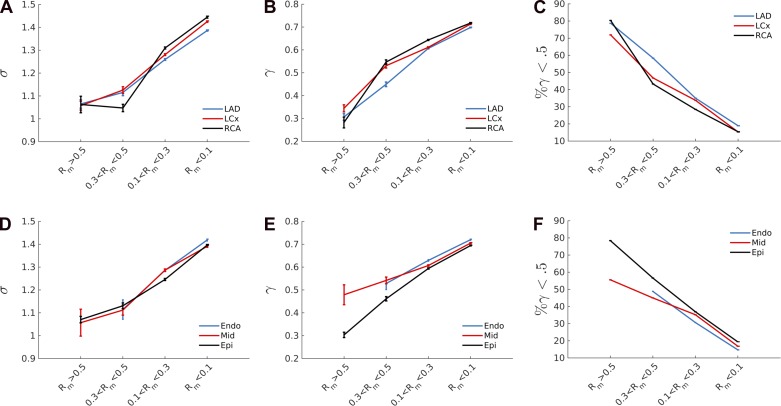
Means and standard errors of the area ratio, σ (*A* and *D*), symmetry ratio, γ (*B* and *E*), and percentage of asymmetrical bifurcations (*C* and *F*) are shown for each vessel cluster. It is clear that as vessel radius decreases, these three variables increase. The vasculature has been classified in terms of mother vessel radius, perfused territory, or transmural location. Interestingly, no difference was found when comparing the same vessel size in different perfused territories or transmural locations.

There is a statistically significant (*P* < 0.01, Wilcoxon signed-rank test) increase in the area ratio (from 1.05 to 1.4) and symmetry ratio (from 0.3 to 0.7) moving from the large epicardial vessels to the small arteries and arterioles, as shown in Fig. 7. Furthermore, the percentage of asymmetric bifurcation (γ < 0.5) strongly decreases from ≈80% in the large vessels to ≈15% in the smaller ones (Fig. 7, *C* and *D*). However, note that there is no statistically significant difference in area ratio and symmetry ratio when comparing different perfused territories (Fig. 7, *A*–*C*) or transmural location (Fig. 7, *D*–*F*) within the same vessel size range. These findings remain unchanged when the *R*_*m*_ interval divisions are adjusted, because changing the mother vessel radius ranges up to 20% causes only a minimal variation (<5%) in the metrics analyzed.

### Validation of Theory

**Fig. 8.**
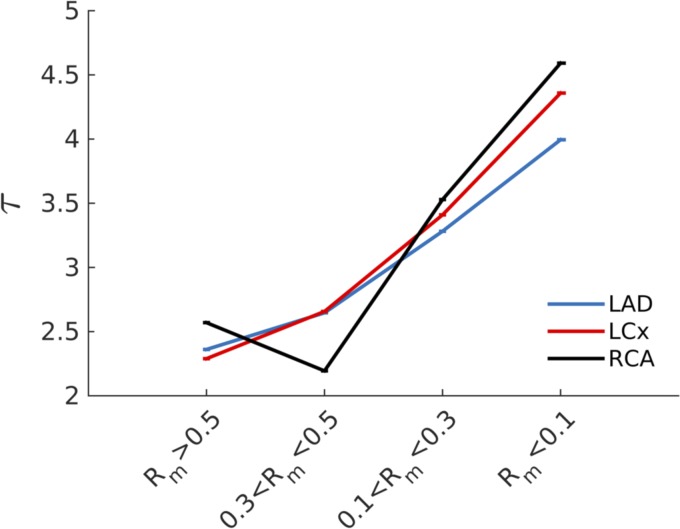
As the mother radius diameter decreases, the scaling law coefficient, τ (see *Eq. 2*), increases. This estimation of τ acquired under the assumption of steady flow regime implies that different scaling law coefficients may pertain to different vascular regions.

The scaling law coefficient, τ, was fitted using the spatial distribution of σ and γ across the coronary vasculature (*Eq. 16*). The fitting process was repeated 50 times for each cluster region, and the solution, τ, providing the minimum mean squared error was chosen. As Fig. 8 clearly shows, there is a statistically significant (*P* < 0.01) increase in the scaling law coefficient (τ) moving from large (*τ* ≈ 2.25) to small (*τ* ≈ 3*.*5) vessels, meaning that different scaling laws pertain to a different scale of vessels. The increase in τ was monotonic in all cases except in the RCA, possibly due to the sparsity of data in that branch. Once more, no significant differences have been found when comparing the LAD, LCx, and RCA perfused territories.

**Fig. 9.**
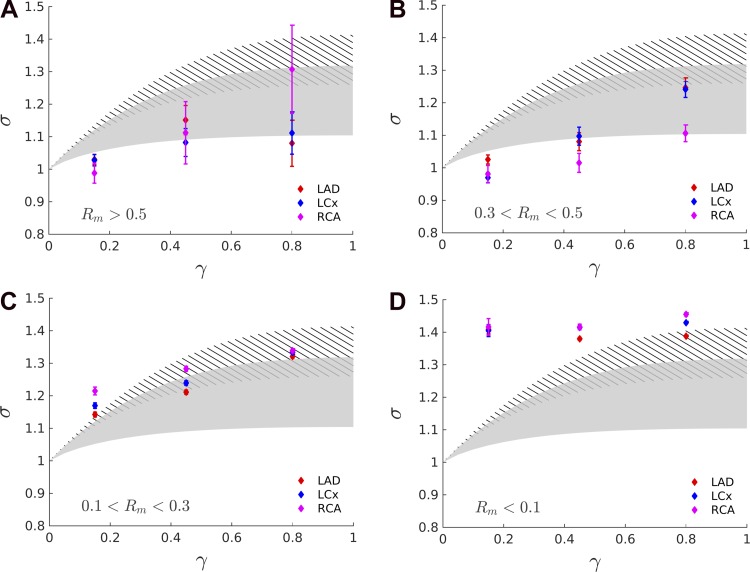
The area ratio-symmetry ratio relationship for the Womersley number range typical of the coronary vasculature is shown for the HK law (shaded) and Murray's law (stripes) overlapped, for the different mother vessel radii (*R*_*m*_) intervals considered. First, the theoretical prediction of higher area ratios for higher symmetry ratios is confirmed by the measurements up to a scale of *R*_*m*_ > 0.1 where the data exhibit a plateau of σ ≈ 1.4. When the varying degree of pulsatility is taken into account, the HK law satisfactorily agrees with the data for the large, medium, and small vessel (*A*–*C*) range being considered. Moreover, it predicts an increase in the σ-γ relationship due to a decreasing Womersley number, which is a consequence of decreasing vessel radius. The theoretical predictions for Murray's law (striped area) do not match the measurements for the large and medium scales.

To validate the proposed theory of combined scaling law and wave propagation, the predictions of *Eq. 23* are compared here with the measurements for Murray's law and HK law, taking into account the effect of varying degrees of pulsatility as captured by the Womersley number. In Fig. 9, the range of area ratio symmetry ratio relationships for the Womersley number typical of the coronary vasculature is displayed for the HK law (gray shading) and Murray's law (stripes). Note that the lower end of Murray's law (α > 10) overlaps with the upper range of HK (α < 1). Note also that the theoretical prediction (see *Eq. 23*) of higher area ratio for higher symmetry ratio is confirmed by the measurements, in the scale *R*_*m*_ > 0.1 where the data exhibit a plateau of σ ≈ 1.4. The implication of this result will be further explored in the discussion. When including the effect of pulsatility, the model prediction for the HK law can accommodate the range in which the data are found for large, medium, and small vessel scales considered (see Fig. 9, *A*–*C*), thus supporting the well-matchedness hypothesis of the coronary bifurcations. Moreover, the theoretically predicted increase in the symmetry ratio for a given area ratio is confirmed by the experimental measurements, in line with the decrease in Womersley number expected in the smaller vessels.

For Murray's law, the theoretical predictions do not match the measurements for the large and medium scales (see Fig. 9, *A* and *B*). For the small scales (see Fig. 9*C*), Murray's law fits the measurements but underestimates the expected Womersley number because for that range of vessels, the expected α [from Kassab et al. (28)] is ∼0.1, which is at the top edge of the striped area.

To summarize, when the pulsatility of flow was disregarded in the analysis of coronary branching patterns, the analysis concluded that different scaling laws apply to different vessel scales (see Fig. 7). However, when the theoretical framework of scaling law was extended to incorporate the Womersley number, the HK law satisfactorily agreed with the anatomical data for vessels of radius larger than 0.1 mm. The same conclusions were obtained when the vasculature was clustered according to the transmural depths, although the details are not shown here.

### Reflection Coefficients Across the Vasculature

**Fig. 10.**
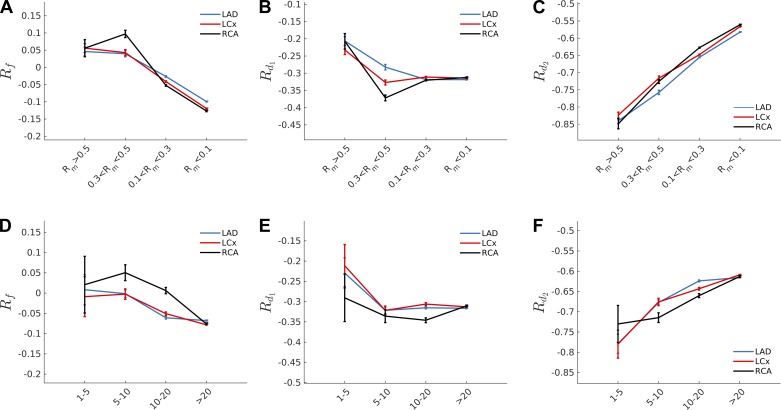
The wave reflection coefficient for the forward-traveling (*R*_*f*_) and backward-traveling waves (*R*_*d*1_,_*d*2_) were calculated for the uniform β* hypothesis clustering the vasculature in terms of mother vessel radius, *R*_*m*_ (*A*–*C*), and Weibel generation number (*D*–*F*). Note that the Weibel generation number starts from 1 at the first bifurcation and increases by 1 at each consequent bifurcation. Similar results were obtained using the hypotheses of uniform pulse wave speed and the experimentally fitted formula (45). Note that in the range of *R*_*m*_ > 0.1 the forward reflection coefficient is approximately zero and supports the well-matchedness hypothesis of the coronary bifurcations. Moreover, smaller vessels were found to exhibit less negative reflection coefficient in the smaller daughter vessel, *R*_*d*2_, at the expense of the larger daughter for which *R*_*d*1_ became more extreme.

The forward (*R*_*f*_) and backward (*R*_*d*1_, *R*_*d*2_) reflection coefficients were calculated for each mother vessel radius, perfusion territories, or transmural location for the three parametrizing approaches described in *Validation of the Unified Framework* (uniform PWS, uniform β*, or an experimentally fitted relationship). The outcomes were somewhat surprising in that the proximal *R*_*f*_, *R*_*d*1_, and *R*_*d*2_ were largely similar (<10% variation) for all parametrization scenarios despite the fact that the assumed distribution in the PWS/β* varied significantly. Moreover, similar results were observed when clustering the bifurcations in terms of the mother vessel radius (*R*_*m*_) or the Weibel generation number (29). As shown in Fig. 10, *A*–*D*, for the constant β* assumption, the forward reflection coefficient is small (*R*_*f*_ ≈ 0), thus once more supporting the well-matchedness hypothesis of the coronary bifurcations for vessels with a radius *R*_*m*_ > 0.1. Smaller vessels have a slightly more negative forward reflection coefficient of approximately −0.1. Moreover, because of the observed increase in symmetry ratio and area ratio for smaller vessels, the backward reflection coefficient *R*_*d*1_ of the larger daughter vessel becomes more negative, whereas the smaller daughter vessel coefficient becomes less negative. This is in good agreement with the theoretical predictions displayed in Fig. 5.

### Species Comparison

**Fig. 11.**
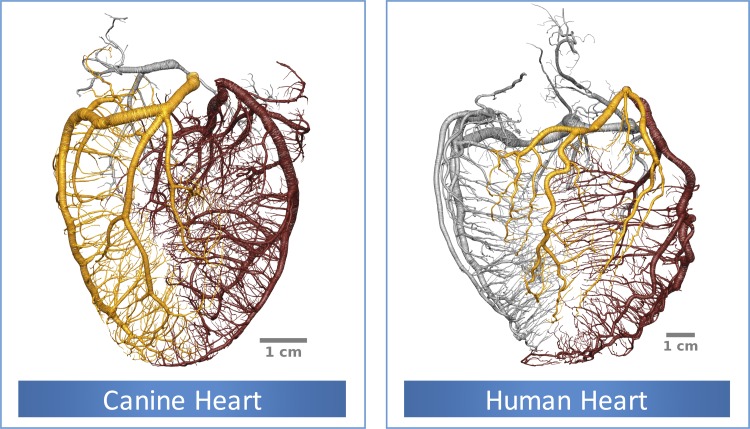
Extracted canine and human coronary vasculatures. The LAD, LCx, and RCA perfused territory are highlighted in brown, gold, and silver respectively. Note that the human vasculature is more tortuous than the canine and porcine coronary vasculature.

The analysis performed above was then repeated for canine and human vasculatures (Fig. 11) to assess possible interspecies variation. Although the superficial appearance suggested a potentially different relationship (the human coronary vasculature is more tortuous compared with the canine and porcine counterparts), the same qualitative trends and quantitative results were confirmed both regarding the σ, γ distribution across the vasculature and the satisfactory prediction of the σ-γ relationship by the HK law, when the effect of pulsatility was taken into account.

## DISCUSSION

The key outcomes of the current study are as follows: augmentation of the scaling laws with the wave transmission theory enabled the implications of specific scaling laws to be studied in an integrated manner in terms of bifurcation morphology and wave reflections. The consequent validation using the pulsatile regime model offered a plausible explanation for the heterogeneity of branching morphology that was previously reported in the literature (12, 52, 77). More importantly, the theoretical results and experimental data supported the hypothesis that the forward well-matchedness is a salient feature of coronary vascular structure. Furthermore, this observation has been shown to be robust to different assumed distributions of vessel material properties and PWS that have been trialed. This in turn implied that the backward well-matchedness is selective; that is, transmission is preferred only from the larger of the daughter vessels and suppressed for the waves traveling up from the smaller daughter vessel. In the discussion below, we examine the theoretical and experimental aspects of the current work and the physiological implications of our findings in greater detail.

### What New Findings Does the Proposed Framework Offer?

In this work, the unification of scaling laws and wave transmission theories led to a demonstration that assessing the well-matchedness of the vessel branching pattern is equivalent to assessing the validity of a specific scaling law. It was shown that recasting the mathematical formulations of both frameworks in terms of area ratio σ and symmetry ratio γ (see *Unified Framework*) provides the same σ-γ relationship (*Eq. 16*) under the assumption of zero reflection coefficient for forward-traveling waves. This is crucial for two main reasons. First, it enables the study of these two aspects in an integrated manner allowing assessment of the implications of a specific scaling law with respect to the distribution of PWS and material properties across the vasculature and vice versa. Second, for each scaling law it provides (under the assumption of well-matchedness) an analytical relationship (*Eq. 16*) that is solely dependent on the anatomical measurements without the need for measuring PWS or β*, thus facilitating validation. Furthermore, the effect that varying Womersley number has on the reflection coefficient at bifurcations has been investigated for the first time in the coronary vessels, which showed that for smaller vessels a decrease in Womersley number causes an increase in area ratios necessary to maintain well-matchedness. Similar observations have been reported in systemic arteries (3).

For validation purposes, two main predictions can be deduced from the newly introduced theoretical framework and compared with the experimental measurements. First, more symmetrical bifurcations (larger γ) have higher area ratios (larger σ), as shown in Fig. 5*A*. Second, smaller vessels have higher area ratios for a given fixed symmetry ratio consistent with the expected reduction in Womersley number (see Fig. 5, *C* and *D*). Both predictions are confirmed by the high-resolution measurements (of around 80,000 bifurcations with a 40- to 60-μm minimum vessel diameter, see Fig. 9).

### Are the Existing Scaling Laws Valid?

The analysis excluding the influence of pulsatility has called into question the validity of a single scaling law applied to the entire vessel network. When the scaling coefficient τ was fit, different values for different vessel sizes were obtained (see Fig. 8), suggesting that the scaling relationship itself may depend on vessel diameter. This dependence of the scaling law parameter τ on the diameter of vessels has been experimentally investigated and reported in the literature. Specifically, VanBavel and Spaan (67) found τ to be 2.82, 2.5, and 2.35 for a mother diameter of <40, 40–200, and >200 μm, respectively. Moreover, the decrease in τ for an increase in the vessel radius (moving from small to large vessels) has been confirmed by other studies (1, 67). These results compare well with our findings (Fig. 8).

On the other hand, the full analysis, including pulsatility, demonstrated the possibility that the HK law is compatible with the measurements when *R*_*m*_ > 0.1, if one takes into account the range of Womersley numbers found in vivo. This further implies well-matchedness throughout the network, according to our analysis. This is because the Womersley regime acknowledges the heterogeneous variation of physiological σ-γ relationships (Fig. 5) that are dependent on the local hemodynamic conditions, rather than demanding a single curve corresponding to nonoscillatory conditions. However, note that we have examined only the branching pattern aspect of the scaling law. Similar variability in the scaling relationships regarding other morphometric parameters (e.g., tissue volume-vascular volume relationship) have been recently examined by van Horssen et al. (69c) and remains a task for future exploration.

To summarize, even when pulsatility was taken into account, the HK law satisfactorily fitted the data, and this implies well-matchedness for coronary bifurcations. However, it is important to underline that the conclusions drawn so far are valid down to a vessel scale of *R*_*m*_ ≈ 0.1 mm. In fact, as can be seen in Fig. 9, for smaller vessels (particularly asymmetrical ones), the measurements do not fit the theoretical predictions. This may be because the anatomical reconstruction at this scale is subject to greater error because the vessel dimension approaches the image resolution limit. However, it is also likely that wave propagation, on which the newly developed theoretical framework is based, plays only a minor role at this scale. In fact, the vessel segments with *R*_*m*_ < 0.1 mm have an average length of ∼14 mm, and a PWS derived from our analysis of approximately 20–25 m/s (depending on the parametrization approach assumed). This implies that a wave would travel through a vessel in less than 1 ms (not measurable by existing techniques and which would require a sampling frequency > 1 KHz). In this regime, the previously proposed Windkessel approximation (i.e., infinite PWS) of the coronary vasculature may be more appropriate (30).

Finally, it is important to recall once more that each proposed scaling law implies a specific branching pattern that has never been evaluated in terms of its consequence to wave propagation. Specifically, once a certain scaling law is assumed, the consequent forward and backward reflection coefficients can be calculated at each bifurcation thus enabling the wave reflection pattern to be assessed.

### What Does the Current Evidence Show Regarding Coronary Wave Reflection Properties?

The validation of the newly introduced theoretical framework supports the hypothesis that coronary bifurcations are well matched for forward-traveling waves, whereas the backward-traveling waves are significantly damped, increasingly so at smaller scales (*R* < 0.3 mm). This conclusion can be derived first by the observation, in Fig. 9, that the anatomical measurements in vessels with *R*_*m*_ > 0.1 mm agree with the theoretical predictions that have been obtained under the assumption of well-matchedness. Second, the reflection coefficient for forward-traveling waves has been found to be *R*_*f*_ ≈ 0 for the analyzed scales, regardless of the parametrization approach used (constant material properties, constant PWS, or experimental formula), with no significant differences in terms of transmurality or perfused territories (see Fig. 10).

The well-matchedness for forward-traveling waves subsequently implies ill-matchedness (strongly negative reflection coefficients *R*_*d*1,*d*2_) for at least one of the backward-traveling waves at the junction, especially for more asymmetrical bifurcations (Fig. 10).

It is noteworthy that the distribution of the forward and backward reflection coefficients has been calculated using the linearized formula derived from *Eq. 17* instead of *Eq. 23*, which takes into account pulsatility. This choice was motivated by the need to minimize the number of assumptions required for calculating the reflection coefficient at each bifurcation (see *Validation of the Unified Framework*). In fact, *Eq. 17* assumes a priori the distribution of PWS (or material properties β*) across the vasculature. Using *Eq. 23* would additionally require the assumption of Womersley number distribution across the network [or to derive it from previous publications (28)]. The fact that different PWS distributions led to the identical outcome of well-matchedness for forward-traveling waves strongly suggests that this is a salient feature of the coronary vasculature. Furthermore, this conclusion is in good agreement with results reported by the Huo and Kassab (22) model of pulsatile blood flow in the entire coronary tree, in which it was observed that the damping of the pressure wave form is rather small for larger vessels and becomes more pronounced for vessels smaller than 100 μm. However, neither the reflection coefficient distribution nor the PWS distribution across the vasculature has been reported, thus a direct comparison with our current results is not possible.

### How Are These Results Relevant to Clinical Challenges?

Wave forms in the coronary vessel have been studied for their clinical diagnostic potential (62, 63). Many recent investigations have employed techniques such as wave intensity analysis (47), which permits specific features of the wave to be identified and ascribed to the spatiotemporal sequence of the coupled cardiac-coronary cycle and their deviation from the norm under diseased conditions. In particular, the role of the backward expansion wave (BEW) has received attention because of its alteration in hypertrophic cardiomyopathy (8), aortic stenosis (7), heart failure (34), and prognosis following myocardial infarction (9) from which novel insights regarding the specific pathology have been derived.

However, although the origin of forward-traveling waves is reliably ascribable to the ejection/suction of the left ventricle, the mechanisms underlying backward-traveling waves have yet to be fully described, thus limiting the conclusions derived by coronary WIA. Specifically, in early clinical research studies, the origin of backward-traveling waves has been putatively ascribed at the microcirculatory level following the relief of systolic myocardial compressions (7, 8, 17, 34, 55). However, there is no specific evidence quantifying the depth of the network at which such relaxation is taking place to give rise to the detected waveforms—in other words, the location of the horizon of observable backward waves remains unidentified.

A more recent study investigating the variation of the coronary WIA profile during the Valsalva maneuver led to the conclusion that coronary wave energy is directly influenced by cardiac mechanical factors (maximal and minimal left ventricular pressure rate) and poorly related to mean coronary flow (51), thus suggesting the compression/decompression of the intramural vessel as the main mechanism driving the backward-traveling waves. Our current estimates of the backward reflection coefficients (Fig. 10) not only support this finding (because waves generated at the microcirculatory level would hardly reach the inlet of the epicardial vessels because of the strongly negative reflection coefficients *R*_*d*1,*d*2_), but they additionally indicate that the observable wave horizon may be markedly proximal in the coronary network. For example, at *R*_*d*1_ = −0.3, only 70% of the waves from the larger daughter vessel would be transmitted up to the parent at each junction. The smaller daughter would effectively contribute no detectable waves past one or two generations.

These observations motivate a reexamination of the existing physiological and pathophysiological conclusions derived by applying coronary WIA. For instance, the lack of diastolic dominance of flow velocity in the right coronary artery compared with the left main stem, associated with a less prominent BEW, is more likely due to the smaller compressive force on the intramural vessels of the right ventricle than on the coronary microvasculature, as originally suggested (17). More importantly, the prognostic value of the BEW in predicting myocardial recovery postinfarction may derive from its direct relationship with left ventricle contractility more than its capability to assess the integrity of the microcirculation after myocardial infarction (9).

In summary, backward-traveling waves are thought to be generated by the combined effect of myocardial compression/expansion of intramural vessels along with a reduction in resistance of myocardial microcirculation during diastole. However, our analysis indicates a diminished involvement of the microcirculatory scale and an expanded role for intramural vessels in producing the backward-traveling waves that were detectable by a catheter positioned in the large epicardial vessels (LAD, LCx, or RCA). Although these considerations are speculative at this stage, they may stimulate a possible shift in the current thinking regarding the physiological understanding of the BEW and the consequent therapeutic targets for intervention whether it be the vascular scale or the associated myocardial region and should be further investigated. A similar revision has already taken place in the systemic pulse-wave literature in which for a long time it was assumed that the major reflection site of aortic pulse was the iliac bifurcation. This was proven to be wrong in more recent studies when catheter wires were advanced more deeply to reveal that the reflection timing did not vary, contrary to conventional wisdom (6). Specifically, that study demonstrated that compounded reflections of waves in the systemic distal circulation may lead to wave trapping, such that waves that originate in distal sites may never arrive at proximal locations. Alternative investigations and debates involving wave trapping are still continuing at present.

In addition, the well-matchedness of coronary bifurcations in large epicardial vessels is an important feature that must be taken into consideration when designing and implanting a stent or a coronary bypass graft (13). The diameter and the material properties should be carefully chosen to maintain the wave transmission properties, otherwise a significant rise in wave reflection at the top of the coronary vasculature may lead to increased shear stress burden and resistance to flow.

### Relevance for 1D Modeling of Coronary Blood Blow

Although 1D blood flow models have gained increasing importance in modeling applications because of their efficiency and ability to accurately represent wave propagation phenomena (38, 66), the parametrization of the vascular network remains an unsolved challenge. Overcome by the difficulties of directly measuring the parameters, many studies adopt the strategy of manually tuning individual segments to achieve idealized wave behavior. Our proposed theory can be used to assess different approaches for assigning wall stiffness parameters to each segment of the network in simulation studies.

For instance, in the low α regime, imposing a uniform PWS or material parameter β* implies *τ* = 2 and *τ* = 2.5, respectively (3). Substituting these values in *Eq. 16* provides the corresponding σ-γ curves. In qualitative terms this would result, for the uniform PWS case, in a constant value of the area ratio σ for all the possible symmetry ratio values, which is not supported by the experimental measurements. On the contrary, the uniform β* assumption would correctly predict increasing area ratios for higher symmetry ratios.

Although introducing pulsatility (*Eq. 23*) to this analysis bestows correct behavior to the uniform PWS scheme, the uniform β* assumption still leads to a superior fit to the experimental data. Despite these differences, all three approaches predict a similar PWS and β* distribution for vessels of radius larger than ∼0.5 mm. For vessels smaller than this scale, the experimental formula and the uniform β* hypothesis predict a strong increase in the PWS in contrast to the uniform PWS hypothesis, which predicts a decrease in β* across the generations.

### What Are the Major Sources of Experimental Error?

Significant efforts have been made to ensure the reliability of the experimental procedure and vascular extraction pipeline. In our experiments, a strict protocol was followed to minimize the variability in data acquisition, and repeatability was demonstrated by consistent outcomes among the three porcine samples. However, it is possible that adenosine may have had a nonnegligible systematic effect on anatomical measurements. As a vasodilator, adenosine may cause a dose-dependent dilation in the coronary vessels beyond a physiological range, leading to a significant drop in resistance (11, 32, 33). Nevertheless, its effects are strongest in the small intramyocardial vessels (*R* < 50 μm) (11), below the scale at which wave propagation is likely to play a strong role as previously discussed. Most importantly, our analysis is based on the area and symmetry ratios, which depend on diameter measurements in close proximity (adjacent to each bifurcation). Thus it is unlikely that adenosine would have caused a significant bias in our measurements.

As described by van Horssen et al. (69c), vascular reconstruction from cryomicrotome imaging is limited by the degree of penetration of the casting material and by the imaging resolution of the experimental setup. However, previous validation studies have shown that the casting material reliably penetrates vessels down to 10 μm, which is far below the resolution required for this study.

### Limitations of the Study

A previous investigation highlighted that even large changes in the scaling law parameter (τ) led to only a small effect on the cost function of the energy minimization (57). This curtailed the possibility of distinguishing between different scaling laws using experimental data. On the contrary, in our model, changing τ within the range of interest (from $\frac{7}{3}$ to 3) caused, for a given symmetry ratio, a substantial variation in the theoretically predicted area ratio (see Figs. 5 and 9), thus avoiding the ambiguities of the previous analyses.

The validity of applying the Womersley analysis in the coronary vasculature on which this work is based is a matter of possible controversy. In fact, it can be argued that the distance between subsequent bifurcations in the coronary vasculature is not sufficient to achieve the implied (Womersley) velocity profile. The question of the optimal theoretical approach to describe the velocity profile in coronary flow is still under debate in the scientific community as noted in remarks by van de Vosse and Stergiopulos (66), and is hampered by the complexity of measuring it in in vivo settings. Nevertheless, multiple 3D in vitro and in silico studies in the literature have illustrated the benefit of employing the idealized Womersley profile to better describe local hemodynamic conditions and wall shear stress distributions in large epicardial vessels (18, 31). This was corroborated by our study, which demonstrated a better agreement between the theoretical predictions and the experimental measurements (see Fig. 9) by adopting the Womersley analysis.

The analysis in the present work was based primarily around diastolic conditions, consistent with the experimental setup from which the anatomical data were collected. The rationale is that for consideration of general structural design of a vascular network, the first-order effects are to be found in the dominant operating condition (i.e., diastole, during which around 80% of arterial flow occurs). The same logic can be applied to variation of heart rate from resting norm (which may alter the pulsatility) and systolic-diastolic variations (resulting in anatomical changes as well as pressure difference, leading to altered PWS in segments). Nevertheless, it would be instructive for exploration to examine how robust the current findings will remain when the cardiovascular system is subjected to varying loads.

Finally, in our work the effects of vessel tapering and nonlinearities on wave propagation were ignored. It is known that nonlinearity in the pressure-area relationship affects wave propagation and that tapering in the geometrical or material properties generates wave reflections. However, our preliminary numerical simulations (not shown) in single tapering vessels in approximate dimensions of a large epicardial vessel demonstrated that tapering and nonlinearities counterbalance each other and only marginally affect the forward- and backward-traveling wave magnitudes (<5%).

### Conclusion

A new theoretical framework that unifies the wave transmission theory with the scaling law formulation has been introduced and validated for the first time. Including pulsatility in the framework broadened the range of measured bifurcation anatomy that can be explained, compared with existing models.

The validation demonstrated that the branching structure in healthy coronary vasculature is well matched, thus favoring forward wave propagation. This feature holds robustly in the proximal network in several species (porcine, canine, and human) regardless of the various assumptions posed on the distribution of pulse-wave speeds (equivalently, the wall stiffness) among the segments. This showed, conversely, that backward-traveling waves are strongly damped when passing through a bifurcation, thus indicating that the horizon of observable backward waves could be significantly more proximal than previously thought in the coronary vasculature.

In addition, new data providing branching patterns across the coronary vasculature have been described in a high level of detail showing that smaller vessels have higher area ratios, higher symmetry ratios, and a higher numbers of asymmetrical bifurcations than large epicardial ones. However, no significant differences was observed in terms of transmurality or perfused territory (LAD, LCx, or RCA).

## GRANTS

This study was supported by the British Heart Foundation Centre of Research Excellence hosted at the Medical Engineering Centre at King's College London, and the European Commission (FP7-ICT-2007/2013 no. 224495: euHeart). The authors acknowledge financial support from the Department of Health, via the National Institute for Health Research (NIHR) comprehensive Biomedical Research Centre award to Guy's & St Thomas' NHS Foundation Trust in partnership with King's College London and King's College Hospital NHS Foundation Trust, the Centre of Excellence in Medical Engineering funded by the Wellcome Trust and EPSRC under grant number WT 088641/Z/09/Z, and Netherlands Organization for Health Research and Development Grants ZonMw 91105008 and 91112030, and the Dutch Heart Foundation Grant NHS 2006B226. J.P.H.M.v.d.W. was funded by Netherlands Organization for Scientific Research Veni Grant NWO/ZonMw 91611171.

## DISCLOSURES

No conflicts of interest, financial or otherwise, are declared by the authors.

## AUTHOR CONTRIBUTIONS

S.R., N.P.S., and J.L. conception and design of research; P.v.H., J.P.v.d.W., R.W., and A.C. performed experiments; S.R., L.H., M. Sinclair, and J.L. analyzed data; S.R., M. Siebes, and J.L. interpreted results of experiments; S.R. prepared figures; S.R. and J.L. drafted manuscript; S.R., P.v.H., J.P.v.d.W., A.C., M. Siebes, and J.L. edited and revised manuscript; S.R., L.H., M. Sinclair, P.v.H., J.P.v.d.W., R.W., A.C., M. Siebes, N.P.S., and J.L. approved final version of manuscript.

## Appendix A

The functions $M_{0}^{\prime }$ and ϵ_0_ were introduced by Womersley (70) to obtain a more compact expression of the velocity profile in pulsatile flow (44). More specifically, this involves the substitutions

(25) $$J_{o}\left(\alpha y^{\frac{3}{2}}\right)=M_{0}\left(y\right)e^{i\theta_{0}\left(y\right)}$$

(26) $$J_{o}\left(\alpha i^{\frac{3}{2}}\right)=M_{0}e^{i\theta_{0}}$$

where *M*_0_ and θ_0_ are the amplitude and phase of the Bessel function *J*_0_ (${ayi}^{\frac{3}{2}}$). $M_{0}^{\prime }$ and ϵ_0_ are then defined as

(27) $$M_{0}^{\prime }=\sqrt{1+h_{0}^{2}-2h_{0}^{2}\;\cos\delta_{0}}$$

(28) $$\epsilon_{0}=\arctan\;(\frac{h_{0}\;\sin\delta_{0}}{1-h_{0}\;\cos\delta_{0}})$$

(29) $$h_{0}=\frac{M_{0}\left(y\right)}{M_{0}}\;\delta_{0}=\theta_{0}-\theta_{0}\left(y\right)$$
